# Supplementary material for: Growth-limiting drought stress induces time-of-day-dependent transcriptome and physiological responses in hybrid poplar
Source: AoB Plants. 2022 Aug 29;14(5):plac040. doi: 10.1093/aobpla/plac040 (PMC9521483; doi:10.1093/aobpla/plac040)
Supplement: plac040_suppl_Supplementary_Figures [file plac040_suppl_supplementary_figures.pdf]

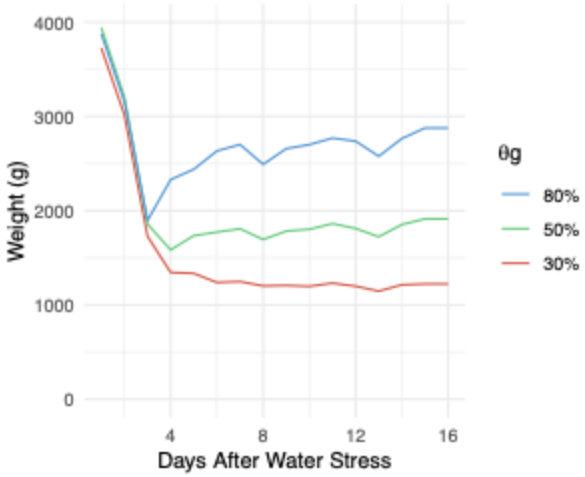

Fig. S1.Daily weights of pots after initiation of water deficit treatments. Once pots had reached their target weights, pots were re-watered to the upper limit of their target weights every morning.

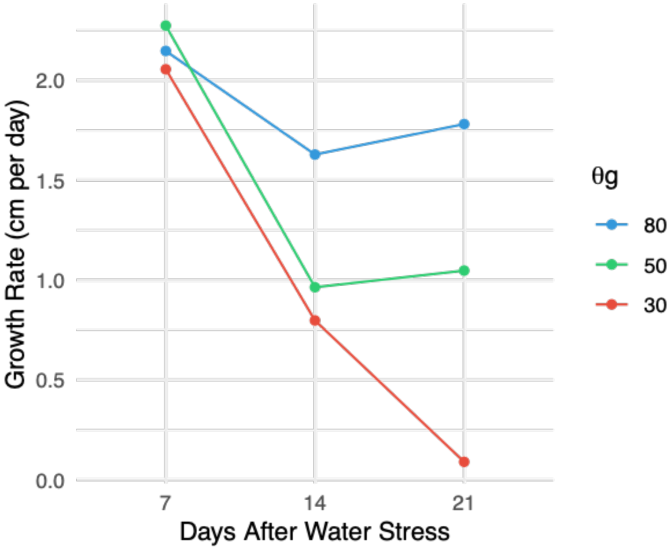

Fig. S2. The growth rate of poplars stabilizes at 14 days after initiation of water deficit, except in 30% SWCg (θg) conditions.

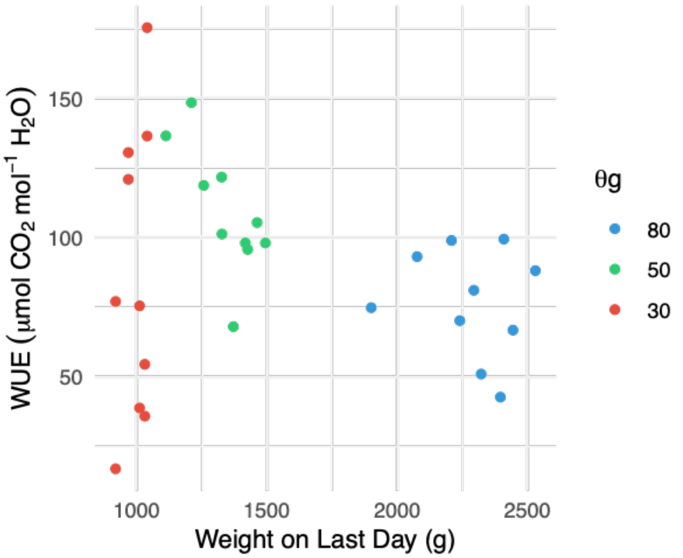

Fig. S3. Water use efficiency (WUE) of 3 SWCg (θg) conditions on the last day of water deficit show variability in the 30% SWCg group.

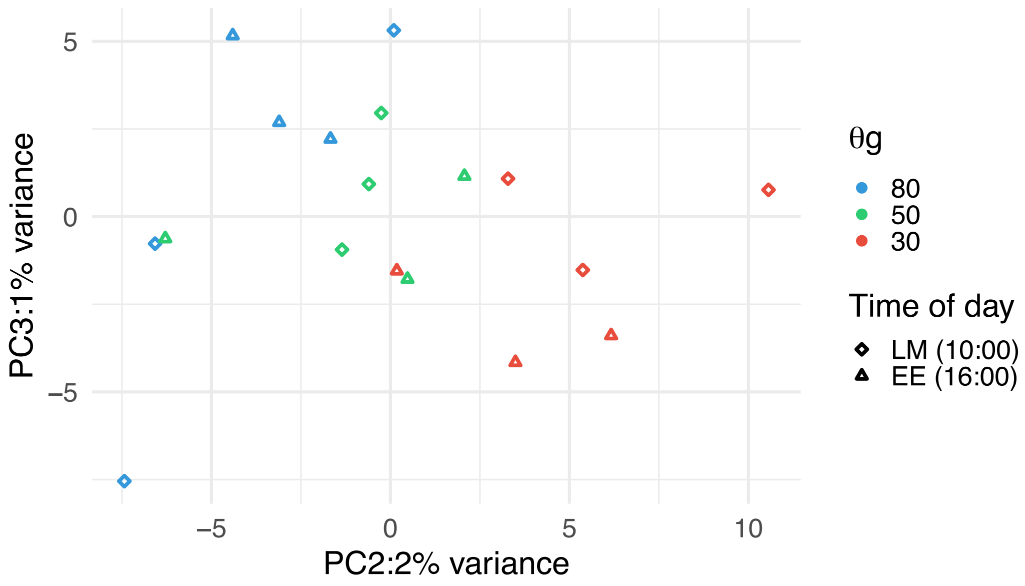

Fig. S4 Principal component analysis of filtered normalized counts for all detected transcripts (23,700 genes). Shape of data point corresponds to time of sampling (diamonds for LM samples; triangles for EE samples); colour corresponds to soil water treatment (blue for SWCg 80%; green for SWCg 50%; red for SWCg 30%). PC1 v. PC2 is shown in Figure 3A.

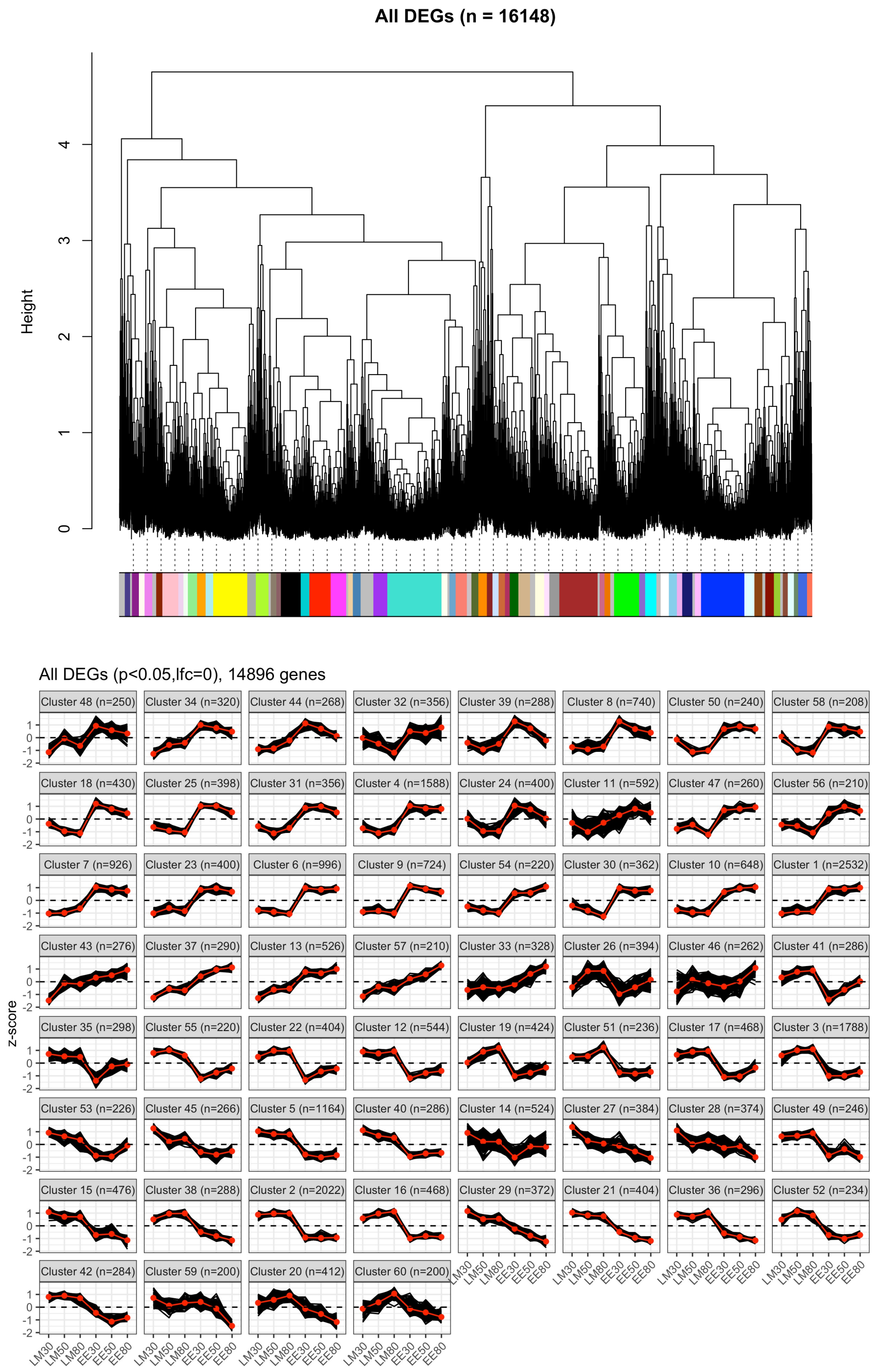

Fig. S5. Hierarchical clustering and expression patterns genes that were differentially expressed between the late morning and early evening (Gene lists in Table S4 and Table S5). The coloured bar below the dendrogram represents the clusters identified by WGCNA. The scaled expression profile of the identified clusters is presented in a line graph with their mean values indicated in

red points and lines. Expression profiles are ordered according to the clusters (coloured bars) from left to right.

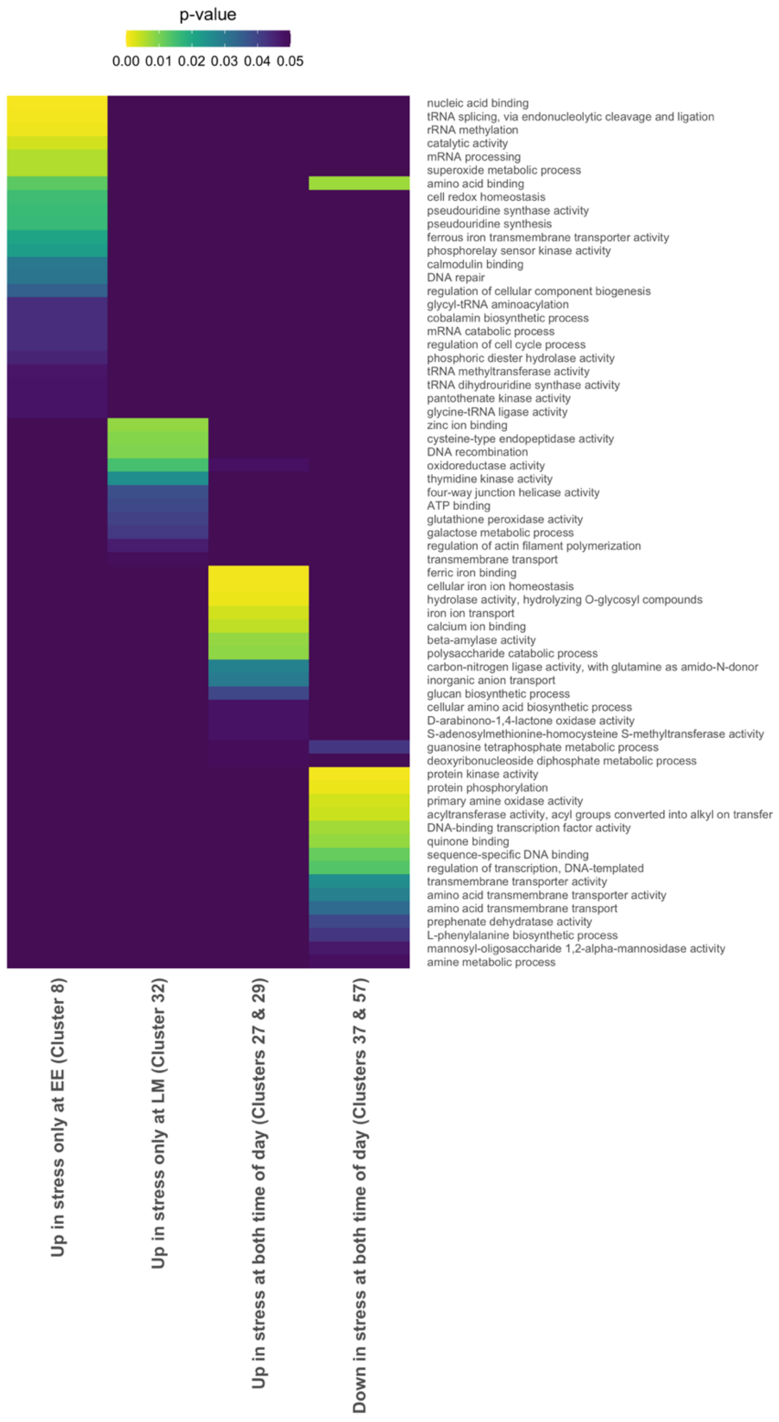

Fig. S6. Selected enriched Gene Ontology terms for gene clustered that responded to water deficit and time of day. Genes in each cluster are listed in Table S5.

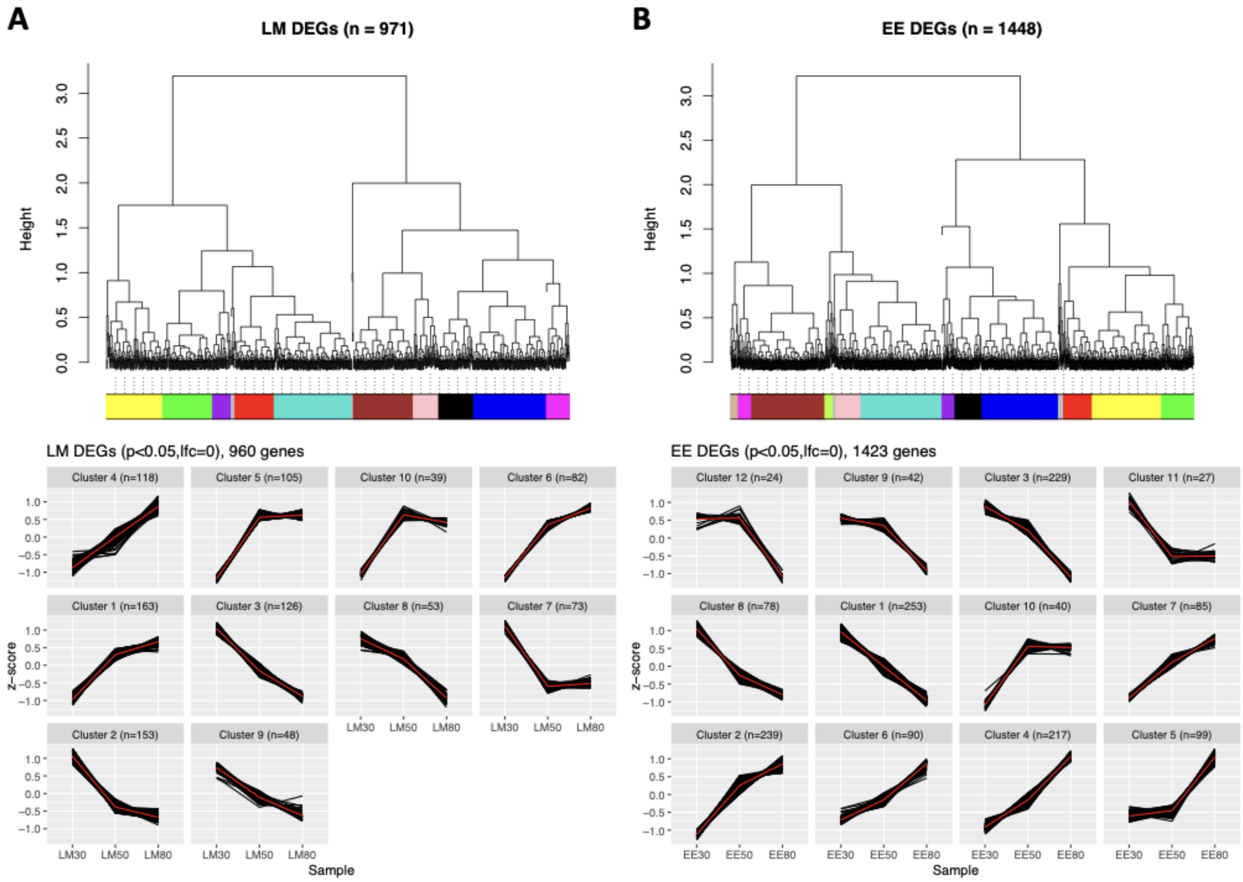

Fig. S7. Hierarchical clustering and scaled gene expression profiles of DEGs in the (A) late morning (LM) and (B) early evening (EE). Expression profiles are ordered according to the colored bars from left to right. Mean expression of the cluster is represented by the red line.

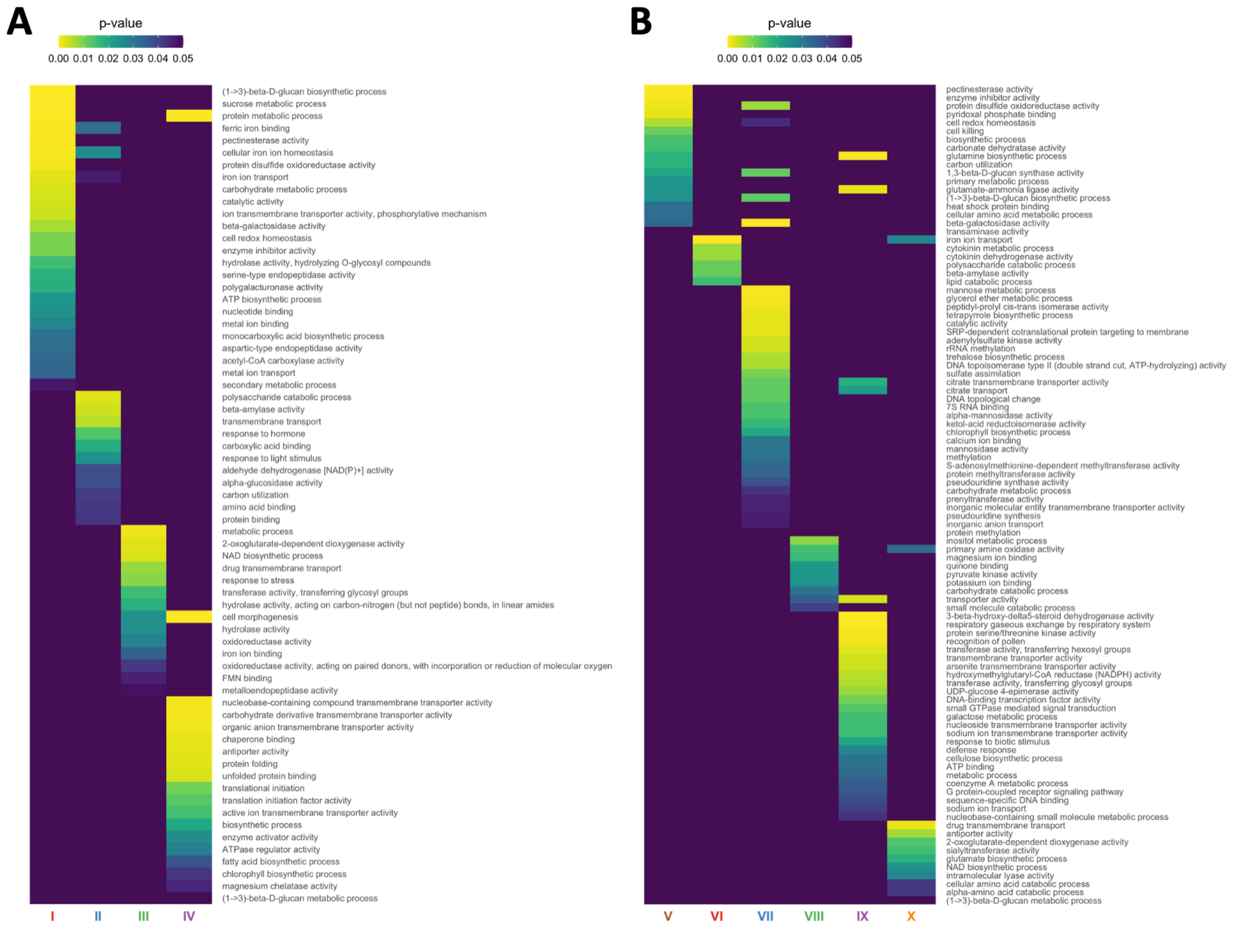

Fig. S8 Full list of Gene Ontology (GO) terms for the (A) late morning and (B) early evening gene expression profiles. See Fig. 4 for list of patterns (I-X).

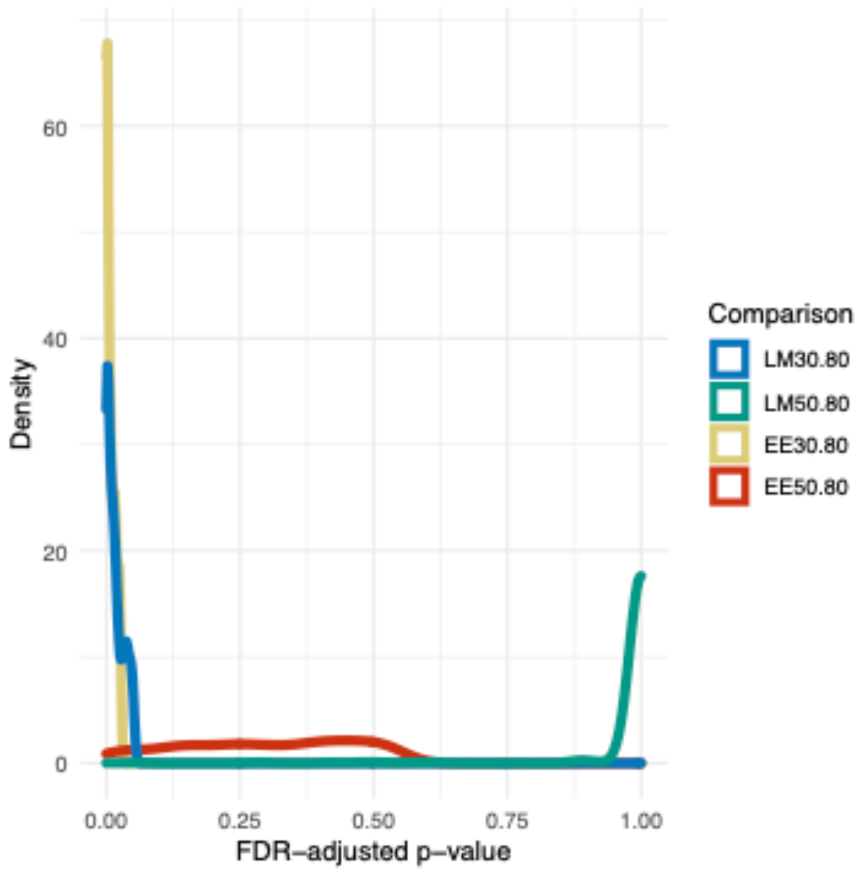

Fig S9. FDR-adjusted *p*-values (BH method) of top 1000 DEGs in each drought treatment (30% and 50% SWCg) compared to the well-watered treatment (80% SWCg) from Fig. 5A.
